# The core interthreshold zone during exposure to red and blue light

**DOI:** 10.1186/1880-6805-32-6

**Published:** 2013-04-12

**Authors:** Naoshi Kakitsuba, Igor B Mekjavic, Tetsuo Katsuura

**Affiliations:** 1 Department of Environment and Technology, School of Science and Technology, Meijo University, 468-8502 Shiogamaguchi 1-501, Tenpaku-ku, Nagoya, Aichi Prefecture, Japan; 2 Department of Automation, Biocybernetics and Robotics, Jozef Stefan Institute, Jamova 39, SI-1000, Ljubljana, Slovenia; 3 Graduate School of Engineering, Chiba University, 263-8522 Yayoi-cho 1-33, Inage-ku, Chiba Prefecture, Japan

**Keywords:** Body temperature regulation, Shivering, Sweating, Core interthreshold zone, Color of light

## Abstract

**Background:**

This study tested the hypothesis that the core interthreshold zone (CIZ) changes during exposure to red or blue light via the non-visual pathway, because it is known that light intensity affects the central nervous system. We conducted a series of human experiments with 5 or 10 male subjects in each experiment.

**Methods:**

The air temperature in the climatic chamber was maintained at 20 to 24°C. The subjects wore suits perfused with 25°C water at a rate of 600 cm^3^/min. They exercised on an ergometer at 50% of their maximum work rate for 10 to 15 minutes until sweating commenced, and then remained continuously seated without exercise until their oxygen uptake increased. The rectal temperature and skin temperatures at four sites were monitored using thermistors. The sweating rate was measured at the forehead with a sweat rate monitor. Oxygen uptake was monitored with a gas analyzer. The subjects were exposed to red or blue light at 500 lx and 1000 lx in both summer and winter.

**Results:**

The mean CIZs at 500 lx were 0.23 ± 0.16°C under red light and 0.20 ± 0.10°C under blue light in the summer, and 0.19 ± 0.20°C under red light and 0.26 ± 0.24°C under blue light in the winter. The CIZs at 1000 lx were 0.18 ± 0.14°C under red light and 0.15 ± 0.20°C under blue light in the summer, and 0.52 ± 0.18°C under red light and 0.71 ± 0.28°C under blue light in the winter. A significant difference (*P* <0.05) was observed in the CIZs between red and blue light at 1000 lx in the winter, and significant seasonal differences under red light (*P* <0.05) and blue light (*P* <0.01) were also observed at 1000 lx.

**Conclusions:**

The present study demonstrated that dynamic changes in the physiological effects of colors of light on autonomic functions via the non-visual pathway may be associated with the temperature regulation system.

## Background

In addition to the pathway of visual sensation, another pathway, the so-called ‘non-visible pathway’, is activated in response to visual stimuli. According to Klein *et al*. [1], visual information received by the retina is transferred to the suprachiasmatic nucleus, and is then further transferred to the paraventricular nucleus of the hypothalamus and to the reticular formation. Finally, it reaches the pineal body, which controls the secretion of melatonin. Many previous studies have demonstrated the effects of light intensity on the central nervous system [2,3] and on the autonomic nervous system [4,5]. Since the pineal body is located near the hypothalamus, which regulates body temperature, reciprocal or one-sided afferent information can be incorporated into the temperature regulation system. Therefore, light intensity would be expected to influence thermal responses such as sweating and shivering.

The core interthreshold zone (CIZ) is defined as the range between core temperature at the onset of shivering and that at the onset of sweating. A constant mean skin temperature (${\bar{T}}_{\mathrm{sk}}$) is required to be independent of thermal responses due to changes in ${\bar{T}}_{\mathrm{sk}}$, and is recognized as reliable information for evaluating the characteristics of body temperature regulation. Kakitsuba *et al.*[6] demonstrated a seasonal difference in the CIZ as well as the effect of light intensity on the CIZ. The latter effect in particular indicates the possible incorporation of the non-visual pathway into the temperature regulation system.

The effect of light color or color temperature on the central and autonomic nervous systems has been extensively studied. According to Takahashi *et al.*[7], the conclusions drawn from experimental studies can be divided into two categories: studies suggesting that red light has an arousing effect as opposed to the mitigating effect of the color blue [3,4,8,9], and studies suggesting that blue light has an arousing effect as opposed to the mitigating effect of the color red [2,10]. Despite the lack of consistent findings on this topic, some effect of light color on the CIZ is expected since body temperature is controlled by the central and autonomic nervous systems. Therefore, the present study tested the hypothesis that the CIZ changes in response to red and blue light exposure.

## Methods

### Subjects

A series of the human experiments were carried out between 2008 and 2010. Five Japanese male subjects aged 22 to 24 years old participated in an experiment conducted during the winter of 2008, 10 different Japanese male subjects aged 21 to 24 years old participated in experiments during the summer and winter of 2009, and 10 other Japanese male subjects aged 21 to 23 years old participated in the summer of 2010.

To estimate the subjects’ maximum work capacity during an incremental load exercise on a cycle ergometer, the subjects were asked to pedal at a rate of 60 rpm, and the work rate was increased incrementally by 10 W/min until the subjects were exhausted or could no longer maintain the required cadence.

All subjects gave their informed consent to participate in the study and were fully aware that they could withdraw from the study at any time without prejudice. The study protocol was approved by the institutional ethics review process.

### Experimental protocol

**Figure 1:**
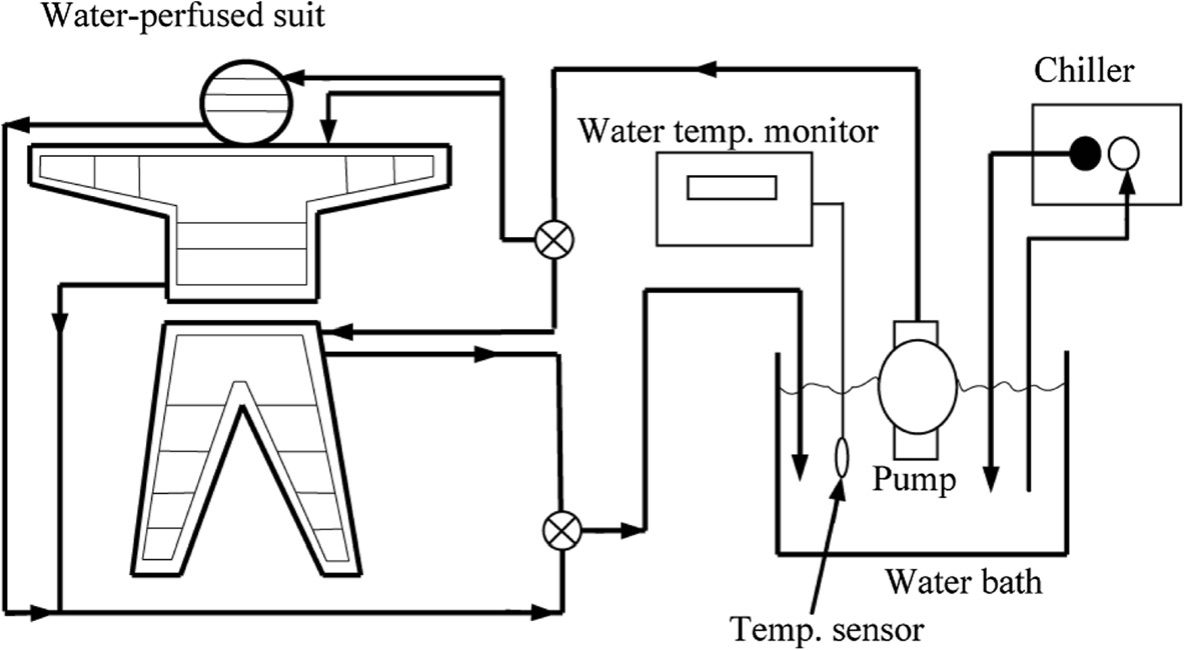
**Diagram of the cooling system.** This diagram appeared in a previous publication (*J Physiol Anthropol* 2007, **26**: 403–409) and is shown again here to provide details of the system. A chiller cooled the water in the bath and a pump supplied cool water into the vinyl tubes incorporated in the suit. After water was perfused through the tubes, it was returned to the water bath and cooled again.

The details of the experimental protocol were as described previously [11]. All the experiments were carried out between 11:00 and 15:00. The ${\bar{T}}_{\mathrm{sk}}$ was calculated from the equation proposed by Ramanathan [12] and was maintained at 28°C by means of a water-perfused suit, described in Figure 1. Subjects wearing water-perfused suits commenced exercising at 50% of their maximum work rate on a cycle ergometer. The exercise was terminated at the onset of sweating, which occurred after 10 to 15 minutes of exercise. The subjects then remained seated on the cycle ergometer for an additional 100 minutes. The onset of shivering was observed when the oxygen uptake started to increase during the last part of the trial, while the ${\bar{T}}_{\mathrm{sk}}$ remained at 28°C. The thresholds were defined as the rectal temperature (T_re_) at which the sweating rate (E_sk_) and oxygen uptake were elevated above the median resting levels.

### Measurements

The T_re_ and skin (arm, chest, thigh and calf) temperatures were monitored with thermistors and the values were stored every 10 seconds using a data logger system (Cadac2 Model 9200A; Cadac, Tokyo, Japan). The E_sk_ was measured at the forehead with a sweat rate monitor (Model SKD-4000; Skinos Co Ltd, Nagoya, Japan). Oxygen uptake was monitored with a gas analyzer (Respiromonitor RM-300i; Minato Medical Science Co Ltd, Tokyo, Japan).

The maintenance of ${\bar{T}}_{\mathrm{sk}}$ while simultaneously extracting 120 W/m^2^ of heat was achieved by having the subjects wear a water-perfused suit (Cool Tube Suit; Med-Eng Systems Inc, Ottawa, ON, Canada). The water perfusing the suit was pumped at a rate of 600 cm^3^/min (Water Pump Model Super Tepcon; Terada, Tokyo, Japan), from a bath in which the water temperature was maintained at 25°C by a Cool Mate Model TE-105 M heat exchanger (Toyo Seisakusho, Tokyo, Japan). A diagram of the cooling system is shown in Figure 1.

**Figure 2:**
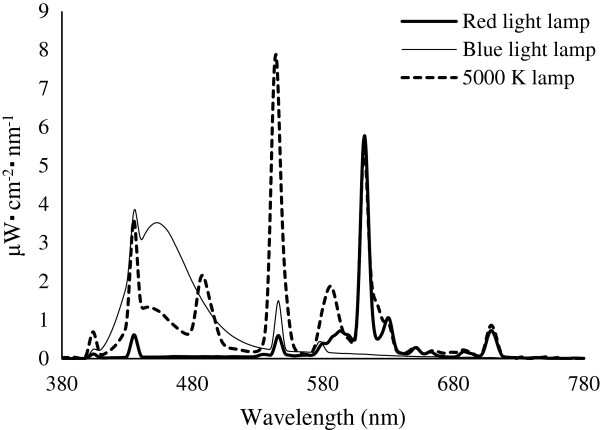
Spectral distribution curves of the red, blue and 5000 K lamps.

In the climatic chamber, light was emitted from red or blue lamps (FLR40S-CR/M and GLR40S-CB/M, respectively; Panasonic Electric Industrial Co Ltd, Osaka, Japan) installed in the ceiling. The spectral distributions of the red and blue lamps were measured using a spectroradiometer (HSR-8100; Maki Manufacturing Co Ltd, Shizuoka, Japan). As shown in Figure 2, the peak emission wavelengths from the red and blue lamps were 612 nm and 436 nm, respectively. The light intensity was measured at the subject’s eye level with a luminance meter (LS-100; Konica Minoruta Co Ltd, Tokyo, Japan). The light intensity was maintained at 500 lx or 1000 lx.

### Statistical analysis

All physiological and psychological variables measured are presented as means ± SD. The CIZs under two light conditions and in two seasons at the same light intensity were analyzed by a paired or unpaired *t*-test. The significance level was set at *P* <0.05.

## Results

The CIZs at 500 lx are shown in Tables 1 and  2. The mean CIZs were 0.23 ± 0.16°C under red light and 0.20 ± 0.10°C under blue light in the summer, and 0.19 ± 0.20°C under red light and 0.26 ± 0.24°C under blue light in the winter. No significant differences between the red and blue lights, and no seasonal differences were observed at 500 lx.

**Table 1:** Core interthreshold zones at 500 lx under red and blue light conditions in the summer

**Subject**	**Red light**			**Blue light**		
	**T**_**re-sw **_**(°C)**	**T**_**re-shivering **_**(°C)**	**CIZ (°C)**	**T**_**re-sw **_**(°C)**	**T**_**re-shivering **_**(°C)**	**CIZ (°C)**
I	37.49	37.2	0.29	37.27	37.07	0.2
II	37.46	37.46	0.00	37.59	37.32	0.27
III	37.46	37.05	0.41	37.43	37.14	0.29
IV	37.41	37.35	0.06	37.35	37.27	0.08
V	37.21	36.87	0.34	37.25	37.05	0.2
VI	37.2	36.77	0.43	37.18	36.8	0.38
VII	37.23	36.95	0.28	36.67	36.52	0.15
VII	36.78	36.58	0.2	36.58	36.36	0.22
IX	37.1	37.07	0.03	36.98	36.93	0.05
X	37.09	36.7	0.39	37.18	37	0.18
Mean ± SD	37.24 ± 0.22	37.01 ± 0.29	0.23 ± 0.16	37.15 ± 0.32	36.95 ± 0.31	0.20 ± 0.10

CIZ, core interthreshold zone; T_re-sw_, sweating threshold; T_re-shivering_, shivering threshold.

**Table 2:** Core interthreshold zones at 500 lx under red and blue light conditions in the winter

**Subject**	**Red light**			**Blue light**		
	**T**_**re-sw **_**(°C)**	**T**_**re-shivering **_**(°C)**	**CIZ (°C)**	**T**_**re-sw **_**(°C)**	**T**_**re-shivering **_**(°C)**	**CIZ (°C)**
I	37.13	37.13	0.00	37.24	37.15	0.09
II	37.59	37.13	0.46	37.41	37.21	0.20
III	37.01	36.48	0.53	36.99	36.58	0.41
IV	37.42	37.37	0.05	36.95	36.84	0.11
V	36.88	36.85	0.03	37.01	36.62	0.39
VI	37.22	37.00	0.22	37.15	36.33	0.82
VII	37.15	36.8	0.35	37.28	36.99	0.29
VII	36.76	36.75	0.01	36.79	36.56	0.23
IX	36.91	36.79	0.12	37.09	37.09	0.00
X	37.24	37.01	0.23	37.19	37.16	0.03
Mean ± SD	37.13 ± 0.27	36.95 ± 0.26	0.19 ± 0.20	37.11 ± 0.18	36.85 ± 0.31	0.26 ± 0.24

CIZ, core interthreshold zone; T_re-sw_, sweating threshold; T_re-shivering_, shivering threshold.

The CIZs at 1000 lx in the summer are shown in Table 3. The mean CIZs were 0.18 ± 0.14°C under red light and 0.15 ± 0.20°C under blue light. The CIZs at 1000 lx in the winter are shown in Table 4. The mean CIZs were 0.52 ± 0.18°C under red light and 0.71 ± 0.28°C under blue light. A significant difference (*P* <0.05) was observed in the CIZs between the red and blue lights at 1000 lx in the winter, and significant seasonal differences were also observed at 1000 lx under red light (*P* <0.05) and blue light (*P* <0.01).

**Table 3:** Core interthreshold zones at 1000 lx under red and blue light conditions in the summer

**Subject**	**Red light**			**Blue light**		
	**T**_**re-sw **_**(°C)**	**T**_**re-shivering **_**(°C)**	**CIZ (°C)**	**T**_**re-sw **_**(°C)**	**T**_**re-shivering **_**(°C)**	**CIZ (°C)**
A	37.08	37.00	0.08	37.20	37.10	0.10
B	37.37	37.14	0.23	37.83	37.15	0.68
C	37.18	36.83	0.35	36.62	36.62	0.00
D	37.08	36.96	0.12	37.15	37.01	0.14
E	37.35	37.28	0.07	37.6	37.13	0.23
F	37.77	37.58	0.19	39.95	36.87	0.08
G	37.15	36.78	0.37	37.04	36.92	0.12
H	37.09	34.77	0.32	37.15	37.08	0.07
I	37.13	37.12	0.01	37.19	37.12	0.07
J	36.76	36.75	0.01	37.53	37.48	0.05
Mean ± SD	3720 ± 0.26	37.02 ± 0.27	0.18 ± 0.14	37.20 ± 0.33	37.05 ± 0.22	0.15 ± 0.20

CIZ, core interthreshold zone; T_re-sw_, sweating threshold; T_re-shivering_, shivering threshold.

**Table 4:** Core interthreshold zones at 1000 lx under red and blue light conditions in the winter

**Subject**	**Red light**			**Blue light**		
	**T**_**re-sw **_**(°C)**	**T**_**re-shivering **_**(°C)**	**CIZ (°C)**	**T**_**re-sw **_**(°C)**	**T**_**re-shivering **_**(°C)**	**CIZ (°C)**
a	37.20	36.66	0.54	37.54	36.89	0.65
b	37.42	36.68	0.74	37.74	36.82	0.92
c	37.05	36.80	0.25	37.48	36.98	0.50
d	37.38	36.88	0.50	37.58	36.62	0.96
e	37.47	36.91	0.56	37.35	36.83	0.52
Mean ± SD	37.30 ± 0.18	36.79 ± 0.11	0.52 ± 0.18	37.54 ± 0.14	36.83 ± 0.13	0.71 ± 0.28

CIZ, core interthreshold zone; T_re-sw_, sweating threshold; T_re-shivering_, shivering threshold.

## Discussion

The results of the present study demonstrated the effects of colors of light on the CIZ during exposure to red or blue light, as well as a seasonal change in the CIZ. The difference in the CIZ under red versus blue light, a seasonal difference in the CIZ under red and blue light, and the CIZ under red and blue light versus 5000 K light, are discussed accordingly.

### Difference in the CIZ under red versus blue light

The CIZs at 1000 lx were significantly (*P* <0.01) wider than those at 500 lx regardless of color of light. This may be due to the effect of light intensity observed under 5000 K light [11]. Although no statistically significant difference in the CIZs was observed at 500 lx between red and blue light, the CIZ under blue light was significantly (*P* <0.05) wider than that under red light at 1000 lx in the winter, suggesting that blue light has a greater mitigating effect on autonomic functions than red light, particularly in the winter. This may be consistent with the results from the studies using red or blue fluorescent lamps, which demonstrated autonomic arousal under red light versus a mitigating effect under blue light [3,4,8,9].

According to the reciprocal cross-inhibition theory by Sherrington [13], the CIZ is a consequence of an elaborate balance of excitatory/inhibitory factors in the sensor-to-effector pathways of heat loss and heat production. If, during the initiation of sweating, exposure to blue light provides a stronger inhibitory effect on the heat loss sensor-to-effector pathway relative to the excitatory effect on the heat production sensor-to-effector pathway, particularly in the winter, T_re-sw_ under blue light would be expected to be higher than that under red light. As shown in Table 4, the mean T_re-sw_ under blue light was 0.2°C higher than that under red light and this was statistically significant (*P* <0.05). This result may support the assumption that change in autonomic function in response to color of light may induce a difference in the CIZ between two colors of light at 1000 lx in the winter.

### A seasonal difference in the CIZ under red and blue light

**Figure 3:**
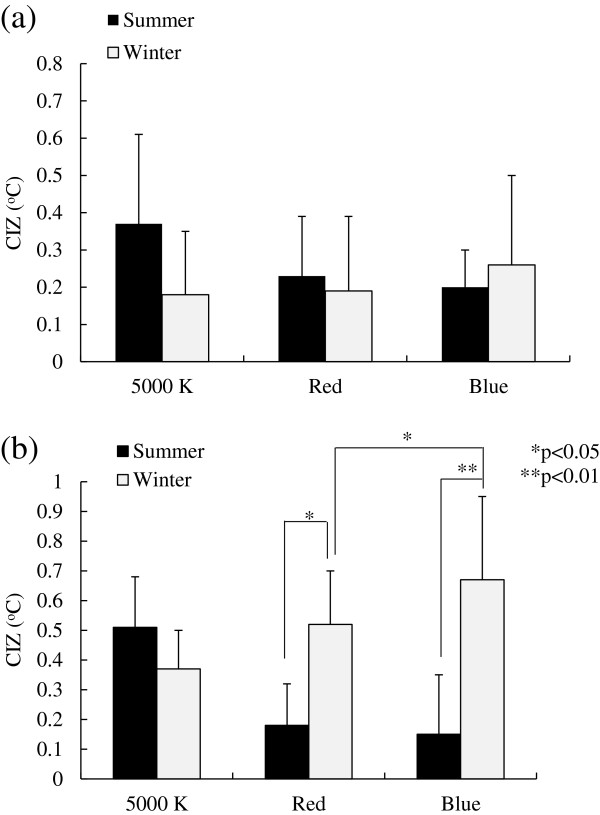
**Change in the core interthreshold zone under different light conditions. (a)** 500 lx and **(b)** 1000 lx.

The CIZs under red and blue light in the winter were significantly wider than those in the summer, unlike the seasonal change in the CIZ during exposure to 5000 K light, as shown in Figure 3b. Since Brück *et al.*[14] demonstrated a difference between the CIZs of cold-acclimated versus cold- and heat-acclimated subjects, Kakitsuba *et al.*[6] reported that the wider CIZ during exposure to 5000 K light may be due to a mitigating effect on the autonomic function as a result of acclimation to both cool and heat in the summer. However, the seasonal change in CIZ under the two colors of light was apparently different, suggesting that some other physiological function may come into play.

The effect of light on human physiology is quite complicated and is not yet fully understood. It is difficult to definitively explain changes in the dynamic functions that affect the sensor-to-effector pathways which are widely assumed to be dependent on season. However, it is interesting to note that the secretion of melatonin is suppressed by an action spectrum [15], which is associated with both light intensity [16] and duration of exposure [17,18]. Thus, the sensor-to-effector pathways may be influenced by an action spectrum.

A seasonal difference under the two colors of light may be based on a variation in autonomic function with season for specific peak wavelengths. If the spectra at 436 nm and 612 nm, that is the peak wavelengths of red and blue light, activate the parasympathetic nervous system in the winter more strongly than in the summer, the CIZ would be expected to widen due to lower T_re-shivering_. As shown in Tables 3 and 4 at 1000 lx, the mean T_re-shivering_ under red light was 36.79°C in the winter, about 0.2°C lower than 37.02°C in the summer. In addition, the mean T_re-shivering_ under blue light was 36.83°C in the winter, likewise about 0.2°C lower than 37.05°C in the summer. This result may support the assumption that seasonal changes in autonomic function, in response to a specific spectrum of light, induce complicated seasonal differences in the CIZ.

### The CIZ under red and blue light versus 5000 K light

Prior to the present study, we carried out experiments under exposure to 5000 K light in the winter and summer using the same subjects who participated in this study [6]. For comparison, the CIZs obtained in that study are shown together with those obtained in the present study in Figure 3a,b. In addition, the spectral distribution curve of the 5000 K lamp reveals that the wavelength of the peak emission is 543 nm with subsidiary peaks at 436 nm and 612 nm, as shown in Figure 2.

Unlike seasonal changes in the CIZ under red and blue light, the CIZ during exposure to 5000 K light is wider in the summer and narrower in the winter, suggesting that the effect of light on autonomic functions is reflected in a difference in the action spectra, since 5000 K light exhibits 543 nm as a peak wavelength. Compared with effects of red and blue light, exposure to 5000 K light in the summer may provide a stronger inhibitory drive, relative to an excitatory drive, at the onset of both shivering and sweating.

According to our previous work [6], the mean T_re-sw_ at 5000 K in the summer was 37.38°C (n = 10), 0.18°C higher than the present results of 37.20°C under red light and 37.20°C under blue light. In addition, the mean T_re-shivering_ was 36.89°C (n = 10), 0.13°C lower than the present results of 37.02°C under red light and 0.15°C higher than the present results of 37.05°C under blue light. These results suggest that the effect of light on autonomic function is reflected in the primary peak emission at 543 nm in the summer.

Compared with exposure to red or blue light, exposure to 5000 K light in the winter may provide a stronger excitatory drive in the heat loss sensor-to-effector pathway, relative to an inhibitory drive in the heat production sensor-to-effector pathway, at the onset of shivering. The mean T_re-shivering_ at 5000 K in the winter, as shown in the previous work [6], was 37.0°C (n = 5), about 0.2°C higher than the present results of 36.79°C under red light and 36.83°C under blue light. These results may also support the hypothesis that the effect of light on autonomic function is reflected in a difference in action spectra in the winter.

The manner of arousing and mitigating effects on autonomic functions against cold or heat stress is summarized in Table 5. Changes are described based on the CIZ at 500 lx. In particular, the effect of 5000 K light as opposed to colors of light can be clearly seen in Table 5.

**Table 5:** The manner of arousing and mitigating effects on autonomic functions

	**5000 K light**	**Red light**	**Blue light**
Summer	⬇	➞	➞
Winter	⬆	⬇	⬇

An arrow ➞ indicates no effect on autonomic functions against cold stress, whereas arrows ⬇ and ⬆ indicate an arousing and a mitigating effect on autonomic functions against cold stress, respectively. On the other hand, arrows  and  indicate a mitigating effect on autonomic functions against heat stress. The latter arrow indicates a greater mitigating effect on autonomic functions against heat stress.

## Conclusions

The results of the present study demonstrate a seasonal change in the CIZ, as well as the effects of light intensity on the CIZ during exposure to red or blue light. It was also confirmed that a light intensity of 1000 lx is necessary to induce a significant difference in the CIZ. To understand changes in the CIZ under various light conditions, we propose that dynamic changes in the physiological effects of colors of light on autonomic functions via the non-visual pathway may be associated with the temperature regulation system.

## Abbreviations

CIZ: Core interthreshold zone; Esk: Sweating rate; Tre: Rectal temperature; Tre-shivering: Shivering threshold; Tre-sw: Sweating threshold; ${\bar{T}}_{\mathrm{sk}}$: Mean skin temperature.

## Competing interests

The authors declare that they have no competing interests.

## Authors’ contributions

NK wrote the manuscript, performed the experiments and analyzed the data. TK and IM were responsible for the coordination of the study, and overseeing data collection and analysis. All authors read and approved the final manuscript.
